# Dataset on the influence of relative humidity on the pathogenicity of *Metarhizium anisopliae* isolates from Thailand and Malaysia against red palm weevil (*Rhynchophorus ferrugineus*, Olivier) adult

**DOI:** 10.1016/j.dib.2020.105482

**Published:** 2020-04-09

**Authors:** Jia Lei Cheong, Wahizatul Afzan Azmi

**Affiliations:** Faculty of Science and Marine Environment, Universiti Malaysia Terengganu, 21030 Kuala Nerus, Terengganu, Malaysia

**Keywords:** Red palm weevil, *Metarhizium anisopliae*, Relative humidity, Bioinsecticide

## Abstract

Red palm weevil (RPW), *Rhynchophorus ferrugineus*, is a polyphagous insect that caused economic damage in various palm species, particularly coconut plantation in Malaysia. Therefore, entomopathogenic fungus *Metarhizium anisopliae* was being introduced in attempts to control biologically the RPW. The entomopathogenicity of an indigenous (Met-Gra4) and foreign (Met-TH) strains of *M. anisopliae* isolated from the soil of Malaysia and Thailand, were tested against RPW adults in laboratory bioassays at 50, 70, 90% relative humidity (RH). Bioassays indicate no significance differences in efficacy between both the conidia of *M. anisopliae* strains against RPW adults. Met-Gra4 showed the highest efficacy at 90% RH (LT_50_ = 6.17 days). However, LT_50_ only slightly differed from Met-TH (6.33 days; 90% RH). Scanning el ectron microscopy for the treated RPWs showed that Met-Gra4 (90% RH) was densely sporulated within the abdomen, while Met-TH can be found mainly across cuticular surface of RPW.

Specifications table

**Table:** 

Subject	Agricultural and Biological Sciences
Specific subject area	Insect Science
Type of data	Table
	Image
	Figure
How data were acquired	Contact bioassay, Scanning Electron Microscope (SEM)- JEOL 6360 LASEM
Data format	Raw
	Analyzed
Parameters for data collection	Contact bioassay on fungal host susceptibility test was conducted at corresponding RH condition at 25 ± 3 °C
Description of data collection	Two different fungal strains (Met-Gra4, Met-TH) were used in the bioassay. Each treatment based of relative humidity (50, 70, 90% RH) was conducted; three treated replicates with five inoculated RPWs per container; three replicates with five uninoculated RPWs as control treatment. Entomopathogen-induced behavior alterations among the inoculated RPWs for each fungal isolates were observed and assessed daily for 2 weeks post-treatment. Data on insect mortality for each treatment within two weeks post-treatment was recorded and analyzed.
Data source location	Fungus laboratory, Central laboratory, Universiti Malaysia Terengganu, 21030, Kuala Nerus, Terengganu, Malaysia
Data accessibility	With the article
Related research article	L.E.L. Grace, M.S.H. Jamilah, M.F. Ahmad, A.A. Wahizatul, Entomopathogenic fungi isolated from the soil of Terengganu, Malaysia as potential bio–pesticides against the red palm weevil, *Rhynchophorus ferrugineus*, Journal of Sustainability Science and Management, 12(2),(2017) 71–79. ISSN: 1823–8556

## Value of the data

• These data provide information regarding geographical regions and fungal habitat condition influencing entomopathogenic fungal efficacy (pathogenesis and epizootiology), in this case, relative humidity that affects fungal germination and host infectivity.
• These data show the potential effect of *Metarhizium anisopliae* MetGra-4 against red palm weevil in Malaysia, as influenced by mycelial growth corresponding to the relative humidity, as the first step to select effective fungal propagule.
• Hypervirulent fungal strains will be applicable in further developing suitable mycoinsecticide formulation to improve their shelf life and enhance its viability in fluctuated environmental conditions for insect pest biocontrol.

## 1 Data description

Two selected virulent strains of *M. anisopliae* which isolated from the soil of Felda Tenang, Terengganu (Met-Gra4) and soil of Muang Chum, Kanchanaburi (Met-TH) were tested against the adults of RPW. The subsequent susceptibility test achieved zero control mortality, which confirmed the entomopathogenic effect of *M. anisopliae* isolates on RPW at three different relative humidity. Overall, at 50 - 90% of relative humidity, data showed that Met-Gra4 required the shortest time period to reach 50% mortality of RPW as compared to Met-TH, of which the shortest time period was achieved at 90% RH (LT_50_ = 6.17 days) (Table 1). However, LT_50_ of Met-TH against the adults of RPW was only slightly longer than that of Meta-G4 which ranged between 0.03 and 0.19 days as RH decreases. Levene's test of equality of error variances indicated that there is insufficient evidence to claim that the variances are not equal $\left(F_{0.05,5,12}=1.785,p=0.191\right)$. In addition, the two-way ANOVA analysis indicated no significant interaction between both the fungal isolates and relative humidity (*p* > 0.05).

**Table 1:** Median lethal time (LT_50_) of adults of RPW treated with two strains *M. anisopliae* isolates (Met-Gra4 and Met-TH) at three different relative humidity.

*M. anisopliae* Isolates	Treatments (Relative Humidity,%)	LT_50_ (Day post-inoculation) (90% CL)*	Equation⁎⁎
Met-Gra4	50	7.20^a^	y=6.9424x
	70	6.48^a^	y=7.7175x
	90	6.17^a^	y=8.0984x
Met-TH	50	7.23^a^	y=6.9162x
	70	6.67^a^	y=7.5007x
	90	6.33^a^	y=7.8947x

LT_50_ followed by the same letter are not significantly different among treatments (*p* < 0.05, Tukey's HSD test).

⁎ Lethal times were determined by probit analysis in SPSS 24.0.0.

⁎⁎ The linear LT_50_ equations of cumulative percentage mortality of RPW in each treatment were calculated based on the slopes.

**Fig. 1:**
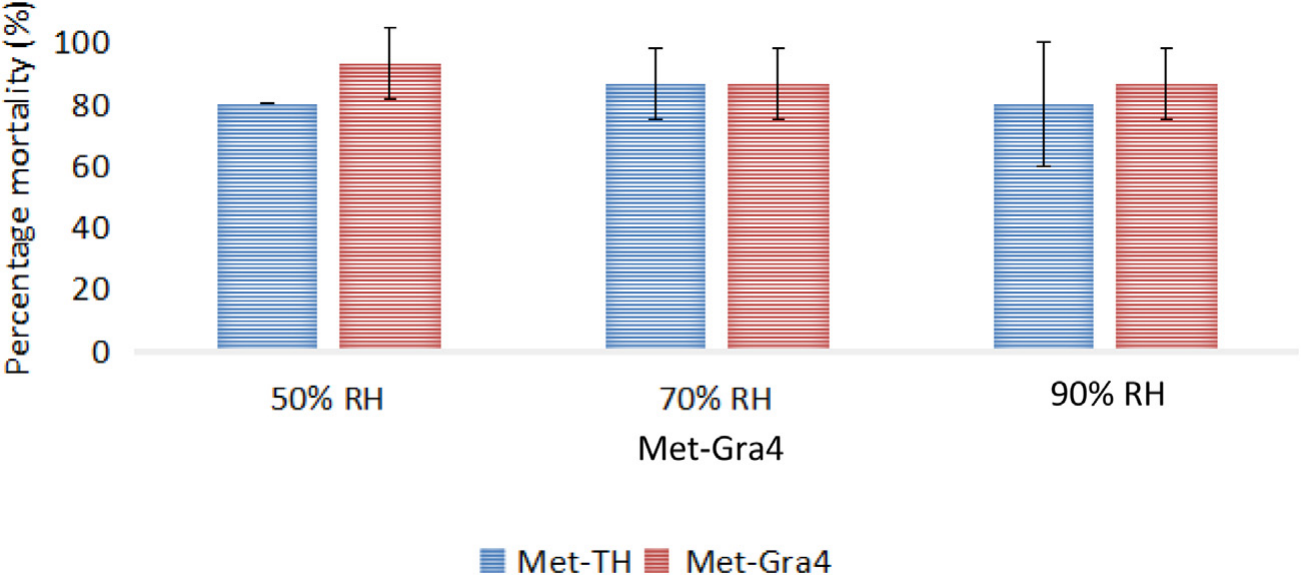
Proportion of fungal infected RPW at different relative humidity.

Subsequently, the actual mortality of treated RPW were determined by observing the mycelial outgrowth of *M. anisopliae*. The observations on the cadavers of RPW illustrated that Met-Gra4 was able to germinate and cause white (initial stage) and green sporulation (late stage) with slightly higher proportion as compared to Met-TH, recorded 93.33% and 86.67%, at 50% and 90% RH, respectively (Fig. 1). However, Met-TH achieved only 80% fungal-induced mortality of RPW adults at both 50% and 90% RH. While the observation on the treatment at 70% RH showed similar percentage of fungal-infected RPW for both *M. anisopliae* isolates.

**Fig. 2:**
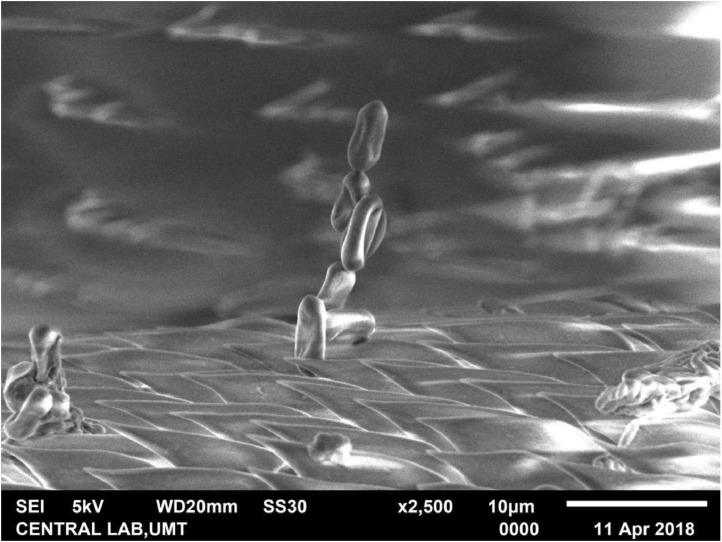
Scanning electron micrograph: adhesion or adsorption of fungal conidia on the scale (hind wing) of RPW.

**Fig. 3:**
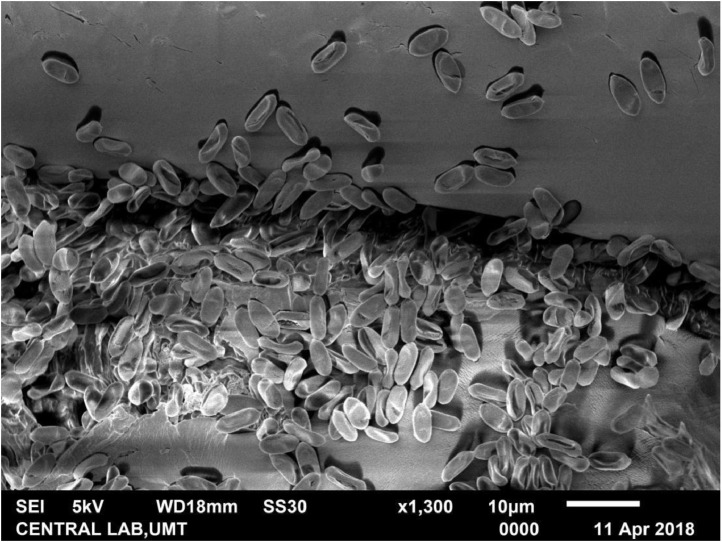
Scanning electron micrograph: adsorption of conidia at the coxal segment of adult RPW (hind leg).

**Fig. 4:**
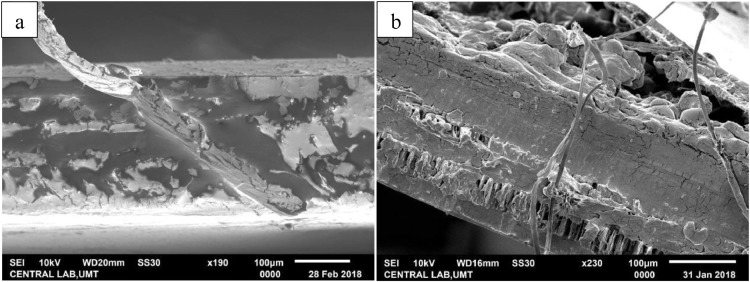
Penetration and establishment of hyphae of *M. anisopliae* isolates in the abdominal cuticle layer (lateral cross section): (a) Met-TH; (b) Met-Gra4.

The fungal-induced mortality of treated adult RPW was indicated by the reddish orange color changes on their cuticle. Scanning electron microscopy (SEM) of adult RPW treated with the fungus *M. anisopliae* (2.0 - 2.8 × 10^8^ conidia per mL) clearly revealed adhesion and penetration structures in the infected adult. Growth relative humidity was at 90%; temperature 25 - 28 °C. Generally, adhesion of the ungerminated conidia for both the Met-Gra4 and Met-TH can be found on the wing scales and the appendage segments of adult RPW (Figs. 2 and 3). The scanning electron micrograph of treated RPW at day nine post-treatment, hyphae of Met-Gra4 was deeply penetrated through the abdominal cuticular layer reaching the inner tissue component (Fig. 4b). In the contrary, Met-TH was found nearly penetrated through abdominal cuticle layer (Fig. 4a).

**Fig. 5:**
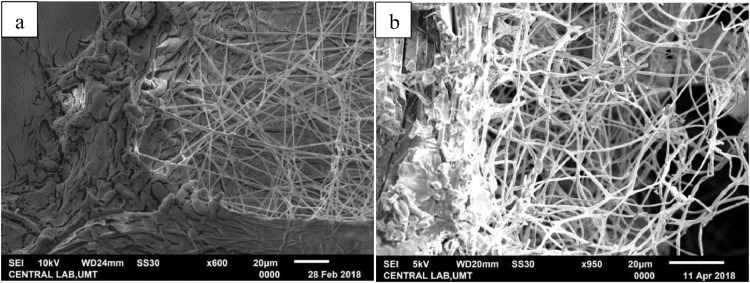
Scanning electron micrograph of fungal hyphal growth within the abdominal region: (a) Met-TH; (b) Met-Gra4.

On the other hand, at day five after the death of RPW, Met-TH as declared by SEM showed a less dense network of hyphal growth within the abdominal region with least decomposed fat and muscle tissues (Fig. 5a). While Met-Gra4 showed a denser hyphal network with more soft tissues being decomposed as indicated by the hollow abdominal cavity (Fig. 5b)

Although fungal-induced mortality of RPW occurred at all humidity level tested, the formation of the characteristic white mycelial growths and conidia only occurred within the 70 to 90% RH ranges (figures not shown). Mycelial growth first appeared at seven days post-treatment. No external fungal growth of any kind was found on cadavers within the 50% RH.

## 2 Experimental design, materials, and methods

### 2.1 Source and isolation of *M. anisopliae*

Hypervirulent fungal strain from Malaysia, named as Met-Gra4 was kindly provided by Grace Lee Ern Lin, Universiti Malaysia Terengganu [1], which was isolated from FELDA Tenang within the latitudes 05° 312′N and longitudes 102° 32′E. While in retrieving fungal strains from Thailand, sampling of agricultural soil (loam to clay loam soil) was conducted in cassava and sugarcane cultivated areas at Muang Chum, Tha Muang district, Kanchanaburi, Thailand. These areas fall within the latitudes 13° 57′54.7″N and longitudes 99° 37′39.4″E. The soil samples from each field were taken from 15 – 20 cm depth below the ground with surface litter removed. Then these soil samples were typically sieved to disintegrate the superficial deposits of gravel, root, grass, litter etc., and to break up large aggregates, and collected in sterile bags. After the sample collection, these were preserved at 4 – 8 °C and stored in dark room until used and processed in the laboratory.

Isolation of EPF was performed by using 10X serial dilution method followed by spread plating of soil sample on artificial media. Soil samples were prepared by first grinding to smaller particles or in a powdery form using sterilised mortar and pestle. About 1 g of each soil sample was diluted in a master tube containing 10 mL of distilled water, and these suspensions were diluted up to 10^−8^ dilution using standard techniques. Afterwards, 10 μm diluted suspensions of 10^−7^ and 10^−8^ dilutions were pipetted and spread on PDA medium plates. Two plates for each soil samples were incubated for 5 – 7 days at room temperature, and based on the morphological appearance, the potential fungal colonies of readily sporulating *M. anisopliae* which characterized by green conidia, were then subcultured by aseptically transferring the inoculum for streaking onto the freshly prepared PDA, plating in Petri dishes, and incubated at room temperature until mycelial growth has appeared. The 5th day after incubation, the fungal culture plates were observed and examined for any sorts of undesirable contaminants, in order to obtain a pure fungal culture. Contemporarily, successive monoxenic subculturing on artificial media recurrently leads to attenuation of fungal virulence. In an attempt to restore fungal virulence after prolonged culture on PDA, each isolate was passed through RPWs prior to culture on plates. Each of the dead RPWs was removed and taken to Petri dishes with moist filter paper-lined bottom, and sealed with Parafilm “M” (Bemis^Ⓡ^, Neenah, WI 54956) to enhance sporulation, as for the use in the consecutive bioassays.

### 2.2 Comparative assessment of the infectivity of *M. anisopliae*

#### 2.2.1 Source of RPW

RPWs were caught by using pheremone traps placed at coconut plantation in/ near Kampung Kubang Badak, UMT campus and Pantai Tok Jembal. In addition, adult weevils that were field-collected using pheromone traps, were baited with high release formulated lures, including ferrugineol (4-methyl-5-nonanol), Ferrolure+ (90% 4-methyl-5-nonanol + 10% 4-methyl-5-nonanone) and kairomone-releasing food bait (pineapple). Captured adult weevils were reared under 20 °C in air-conditioned culture room, placing in plastic rearing containers. Sugarcanes were baited and supplanted twice a week. In the subsequent tests, the field-collected RPWs without any defect or damage, with its body length within the range of 3.0 – 3.5 cm were selected. Mixed-sex adult RPWs were precleaned with running tap water before surface sterilized with 70% alcohol for 10 s, continued by immersing in sterile distilled water 3 times consecutively.

### 2.3 Preparation of conidial suspension

For conidia production, subcultures of *M. anisopliae* on PDA plates were incubated at 28 °C for 2 weeks. In preparing and making initial conidia suspension, the conidia was gently scraped off from the PDA surface by adding sterilised 0.02% aqueous Tween 80^Ⓡ^ solution into the culture plates. The premixed conidia suspensions were then centrifuged at 5000 rpm for 15 min to separate the conidia from the mycelium and other waste [2]. The suspensions were filtered through sterile cheesecloth to remove hyphal fragments, and regularly hand vortexed whenever needed to decimate conidia clumps in order to obtain homogenous mixtures. The concentration of the stock conidial solution was determined microscopically using haemocytometer (Neubauer-improved chamber) with the aid of a compound microscope. Prior to that, appropriate dilutions were performed to achieve reliable conidial count, in order to reduce statistical error. After the concentration of the stock solution had been determined, its concentration was adjusted to 10^8^ conidia mL^−1^ based on the formula below:

$$C_{1}V_{1}=C_{2}V_{2},\;\text{where}$$

*C*_1_ = Concentration of the stock solution, conidia per mL
*V*_1_ = Calculated volume required to be taken out from the stock solution, mL
*C*_2_ = Desired concentration to be prepared, conidia per mL
*V*_2_ = Volume a of desired concentration, mL

### 2.4 Host susceptibility at different relative humidity

Disinfected RPWs were dipped in the prepared conidial suspension for 120 s, providing a dose of 10^8^ conidia/ insect, thereafter inoculated RPWs were maintained in containers at different relative humidity and room temperature. Each container was perforated with only two small holes to minimize gaseous exchange. The deliquescence relative humidity was thermodynamically manipulated using supersaturated salt solutions with pure hygroscopic salt minerals: potassium carbonate (50%), sodium chloride (70%), potassium chloride (90%), in accordance to the experimental value provided [3]. Anhydrous CaCl_2_ and distilled water, provided 0% and 100% relative humidity, respectively. Approximately 100 mL of saturated salt solution was placed within the container according to the treatment. Each RH treatment was conducted using: three replicates with five inoculated RPWs per container, and three replicates with five uninoculated RPWs as control treatment. Entomopathogen-induced behavior alterations among the inoculated RPWs for each fungal isolates were observed and assessed daily for 2 weeks post-treatment. Dead cadavers were surface-sterilised for 3 min in 2.5% NaClO solution to avoid secondary fungal contaminants prior to incubation under darkness with corresponding RH condition at 25 ± 3 °C. Observation and photographic record of sporulating cadavers were conducted for 10 days.

### 2.5 Surface morphological assessment

The surface morphological investigation was in accordance to [4,5] with slight modification. Inoculated RPW was collected ten times post-treatment at 24 h interval (during laboratory bioassays). Specimens were preprocessed: (i) fixed using 2.5% glutaraldehyde in 0.1 M sodium cacodylate buffer, pH 7.2 for 2–4 h, (ii) rinsed 15 min for 3 times in 0.1 M sodium cacodylate buffer, pH 7.2, (iii) post-fixed in 1% osmodium tetroxide in 0.1 M sodium cacodylate buffer, pH 7.2 for 2–4 h, (iv) repeated step (ii), (v) dehydrated the specimens sequentially from 35% - 100% ethanol (EtOH), and each different percent EtOH would take 20 min, (vi) dissect and cut desired parts of RPW, and (vii) sputtered with ultra-thin gold palladium films coating (thickness range of 2–20 nm) using evaporator, in order to reduced SEM beam damage. Dehydrated specimens were examined under scanning electron microscopy (SEM) at low vacuum mode to characterize morphologically the process of infection by *M. anisopliae*. Micromorphology of the *R. ferrugineus* cuticular cross-section was observed and photographed using SEM. High quality reference images of fungal germ tube penetration and development were documented.

### 2.6 Statistical analyses

The mortality mean value of the five replicates for each treatment was used throughout the statistical analysis. The fungal infectivity percentages towards RPW in four different treatments was corrected by eliminating the natural mortality in control treatment (0 − 5%) conforming to the Schneider Orelli's formula:

$$\text{Corrected}\text{efficacy}\%=\left[\frac{\left(\text{treated}\text{mortality}\%\right)-\left(\text{untreated}\text{mortality}\%\right)}{100-\left(\text{untreated}\text{mortality}\%\right)}\right]\times 100$$

Subsequently, the corrected mortality data was subjected to probit analysis to determine the significance for LT_50_ at different treatment of relative humidity. Two-way ANOVA with Tukey's post hoc analysis was conducted to determine whether there were significant differences in the total mortality of RPW between fungal isolates at the different treatments. All tests were carried out by using IBM SPSS Statistics 24.

## Appendix Supplementary materials

Image, application 1

Image, application 2
